# Refractory Rheumatic Disorder: Atypical Postpregnancy Osteoporosis

**DOI:** 10.1155/2015/327965

**Published:** 2015-02-17

**Authors:** Cindy Mourgues, Sandrine Malochet-Guinamand, Martin Soubrier

**Affiliations:** CHU Gabriel Montpied, Service de Rhumatologie, 58 rue Montalembert, 63000 Clermont-Ferrand, France

## Abstract

This is a case report on a young patient with severe osteoporosis that was initially revealed when she presented with polyarthralgia during her second pregnancy. Postpartum, the pain increased and her X-ray did not show any abnormalities. A bone scintigraphy was performed. It indicated an inflammatory rheumatic disorder. Six months after partum, an investigation of right coxalgia revealed a spontaneous basicervical fracture. Given the persistent polyarthralgia, the patient underwent a new scintigraphy, which revealed areas of what looked to be old rib and L1 fractures. A subsequent full body magnetic resonance imaging (MRI) scan revealed signal abnormalities that could indicate multiple lower limb bone fractures. Despite exhaustive biological, radiological, and histological testing, no secondary cause for the osteoporosis was found. The patient was started on teriparatide. We finally concluded that, despite the atypical presentation, the patient was suffering from postpregnancy osteoporosis. It is possible that the frequency of occurrence of this still poorly understood disease is underestimated.

## 1. Introduction

Described for the first time in 1955 by Nordin et al., postpregnancy osteoporosis typically occurs in primiparous women of a mean age of 28 years during their third trimester of pregnancy or in the immediate postpartum period [1]. To date, about a hundred case reports and four case series have been published. Postpregnancy osteoporosis is a rare condition and the cause is poorly understood. It can occur in women who have a low prepregnancy bone density. The rare occurrence of relapse in subsequent pregnancies is an original phenomenon with this condition. The frequency of occurrence is probably underestimated [2]. Joint pain during pregnancy is generally due to mechanical factors and the loosening of muscles and ligaments. We are reporting a case that was complicated by a femoral head fracture after the patient had been initially diagnosed with inflammatory rheumatic disorder.

## 2. Case Report

A 27-year-old female patient was hospitalized for a work-up of a spontaneous right femoral head fracture in March 2013. The patient had a history of moderate tobacco use of less than ten pack-years. The patient has two children (4 years and 6 months of age) whom she nursed 5 months and 1 month, respectively. She also had four miscarriages, one of which occurred in her fourth month of pregnancy. Her BMI is 30 kg/m^2^, but her weight has drastically fluctuated in a span of less than five years.

**Figure 1:**
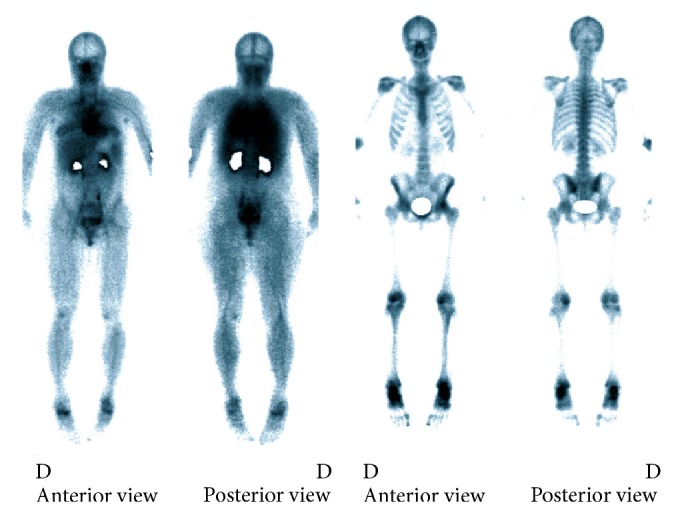
Bone scintigraphy performed two months after partum. Excessive uptake by both tali, bilaterally by the femoral condyles and by the left cotyle.

**Figure 2:**
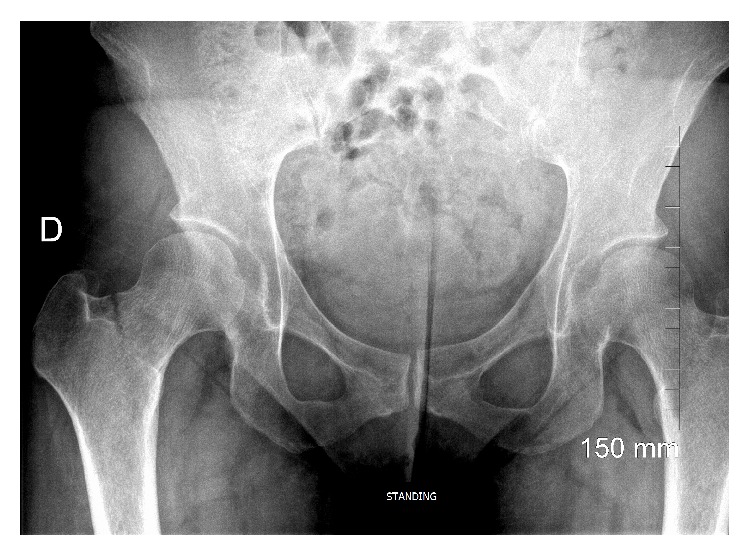
Pelvic X-ray, anteroposterior view. Basicervical fracture of the right femur.

**Figure 3:**
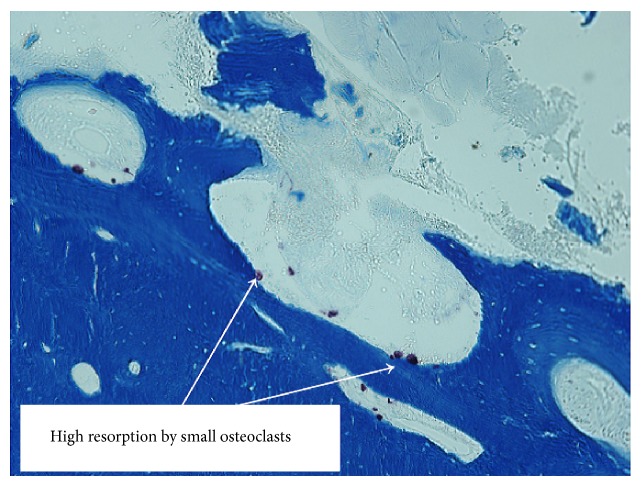
Bone biopsy of the iliac wing with double tetracycline labelling. Active, highly intense resorption via very small osteoclasts, especially in the periosteal region.

Progressively, starting at the beginning of her 2nd pregnancy, she began experiencing daytime and nighttime arthromyalgia anteriorly and bilaterally in her legs. The pain migrated upwards from her knees to her lower thighs. Two months after partum, given the persistence of her pain, she underwent bone scintigraphy. The test revealed abnormal uptake indicating inflammation at each Chopart joint, the left talar dome, the internal left knee, the right femoropatellar joint, and finally the upper left coxofemoral joint (Figure 1). The standard X-rays were normal. One month after these tests, the patient complained of right-sided thoracic pain. Radiological testing was negative. Six months after partum, the patient's coxalgia had worsened, leading her to undergo new standard X-rays that demonstrated a fracture of the right femoral head complicated by a spontaneous basicervical fracture (Figure 2) treated with osteosynthesis. The bone biopsy performed during the surgery did not reveal any abnormal cells. There was no family history of bone disease. Laboratory testing one month after the femoral fracture revealed normal plasma and urinary calcium and phosphorous levels as well as normal parathyroid hormone levels. Her vitamin D was 10.8 ng/L (*N* > 30 ng/L). She had no signs of inflammatory syndrome and her blood protein electrophoresis levels were normal. Her biological examinations ruled out hyperthyroidism, premature ovarian insufficiency, and Cushing's syndrome. Her iron and tryptase levels were normal. Her antitransglutaminase antibody levels were normal. The only abnormal laboratory results were chronically elevated alkaline phosphatase levels of over 150 U/L (*N*: 5–135 U/L). Bone density testing revealed osteoporosis at two sites with a *z*-score of −3.1 SD at the spine and −2.7 SD at the femoral head. Endoscopy ruled out coeliac disease and inflammatory bowel disease. The patient also underwent a CT scan of her chest, abdomen, and pelvis as well as a PET scan. No tumours or lymphadenopathies were detected. However, the CT scan did reveal several old fractures, mainly of the right ribs, although no trauma was mentioned during the history. A new bone scintigraphy revealed intense uptake in several left medial ribs indicating fractures, as well as in L1 even though the patient had never experienced lumbar pain symptoms. Two months following her femoral fracture, given the onset of new pain, especially in the left lower limb, another full-body MRI was performed. The examination revealed signal abnormalities indicative of bone oedema that could mean fractures of the left talus, the internal tibial plateau of the left knee and the left greater trochanter, requiring immobilization. Given the unusual nature of this fracture-inducing osteoporosis, the patient underwent a bone biopsy after double tetracycline labelling. It revealed trabecular and cortical osteoporosis with active, highly intense hyperabsorption, especially subperiosteally, by very small osteoclasts (Figure 3) and normal or accelerated formation indicating coupled hyperremodelling. There was no primary mineralization disorder. Unfortunately, the bone biopsy provided very little information on the trabecular bone due to the poor biopsy quality. In contrast, there were no abnormal cells found or any argument for an overload or mastocytosis. Given the exhaustive negative aetiological work-up, we diagnosed the patient with postpregnancy osteoporosis. Due to the severity of the symptoms and the successive fractures, teriparatide treatment was initiated. Despite this, the patient experienced a new rib fracture without any apparent trauma after six months of treatment.

## 3. Discussion 

We are reporting on a case of fracture-inducing postpregnancy osteoporosis that was initially thought to be an inflammatory rheumatic disorder and became complicated by a femoral head fracture. Our case report is unusual compared with other cases found in the literature due to the type of fractures involved and the fact that onset followed a second pregnancy. The most frequently described fractures occur after an initial pregnancy and are vertebral [3]. However, observations of vertebral fractures have been reported with postpregnancy osteoporosis arising only after a second pregnancy [4]. Postpartum sacral, foot, and rib fractures have also been described [5]. Femoral fractures are generally described as transient osteoporosis or hip algodystrophy that can also become bilateral. Some femoral head fractures have also been described well after the postpartum period [6]. Our patient had no signs indicating algodystrophy on her pelvic X-ray and the presentation was indeed consistent with multiple osteoporotic fractures. Nevertheless, it is interesting to observe that transient hip osteoporosis can also be accompanied by low bone density, thereby complicating the understanding of the pathophysiology of these different postpregnancy rheumatological conditions [6].

The pathophysiology of postpartum osteoporosis is not well understood. Disturbances in phosphorous and calcium metabolism, a decline in bone density during pregnancy, bone remodelling changes, and a possible genetic predisposition given observations of family members are all etiological hypotheses traditionally described in the literature. Standard biological testing often reveals normal phosphorous and calcium levels except for hypercalciuria. Alkaline phosphatases are often physiologically elevated during pregnancy. Pregnancy is also often accompanied by Parathormone Relative Peptide (PTHrp) hypersecretion. PTHrp is a peptide synthesized by numerous nontumoural tissues, including the mammary glands. It plays a key role in maintaining calcium balance in pregnant and nursing woman. It promotes bone catabolism to mobilize calcium for the foetus, especially during nursing, leading to an estimated maternal bone loss of about one percent per month. When breastfeeding, calcium comes only from bone catabolism and not from food intake [7]. There are reports in the literature of postpregnancy osteoporosis associated with hypercalcemia due to persistently elevated PTHrp in a 31-year-old patient despite stopping nursing [8]. The role of PTHrp in postpregnancy osteoporosis has not been thoroughly reviewed. Its level was not determined in the case we are reporting here.

In our observation, the bone biopsy data did not help establish an aetiologic diagnosis. The literature has little histomorphometric data on postpregnancy osteoporosis, and what data are available are disparate. There are many methodological differences and the biopsies were performed at variable times ranging from several months to several years after pregnancy, or even during pregnancy. At times, osteoporosis was confirmed solely based on trabecular bone analysis and at other times based on cortical bone analysis. Bone remodelling seemed normal or exaggerated. In the majority of cases, bone mineralization was normal [9]. The bone biopsy of our patient demonstrated hyperactive subperiosteal resorption that has never been described in postpregnancy osteoporosis. This observation could indicate hyperparathyroidism, but the osteoclastic morphology was unusual and the parathyroid hormone levels were normal.

The treatment most frequently used to treat postpregnancy osteoporosis is bisphosphonates [10].

More recently, teriparatide was offered due to the fear of bone remodelling with bisphosphonates, the passage of these molecules into the placenta, and the potential impact on future pregnancies. Three patients who suffered vertebral fractures six months after partum received teriparatide treatment for six months. They experienced a 14.5 to 25% gain in lumbar spine bone mass, a 9.5% to 16.7% gain in femoral head bone mass, and a 13.4 to 17.9% gain in total hip bone mass, without experiencing any new fractures [11]. In the literature, other treatments have been offered, such as strontium ranelate [12] and even vitamin K2 or menatetrenone, which increases the carboxylation of osteocalcin [13].

Our patient received teriparatide treatment given the delay in consolidation of her femoral fracture and the severity of her fracture-related symptoms.

In our case fractures occurred during postpartum period but musculoskeletal pain has started before. We do not have any idea of the bone density of our patient before and during her pregnancy. Prevalence of osteoporosis during pregnancy is unknown because the main diagnostic methods involve radiation, which is usually avoided in pregnant women [14]. Thus, diagnosis is made often at a later stage. Our patient's observed osteoporotic risk factors were smoking and breastfeeding, although considering the latter as a risk factor is controversial. The risk of osteoporosis due to breastfeeding seems to vary with parity, duration of breastfeeding, and maternal age [15]. The aetiological investigation for secondary osteoporosis revealed only hypovitaminosis D. This condition can induce widespread pain. Kuru et al. found in their study that patients with hypovitaminosis D have a significantly higher pain scores for all scales (*P* value range 0.002) among 83 patients with chronic widespread pain [16]. However, to our knowledge there are no data about pain and hypovitaminosis D during pregnancy. Otherwise in our case, pain was well explained by occurrence of multiple fractures.

## 4. Conclusion

This is a report of a case involving a patient suffering from multiple fractures, the painful symptoms of which initially led us to believe that she was experiencing an inflammatory rheumatic disorder. Exhaustive testing revealed bone fragility but did not reveal a secondary cause of the osteoporosis. Despite the atypical manifestations, we diagnosed the patient with postpregnancy osteoporosis.

Postpregnancy osteoporosis is a rare condition whose pathophysiology is still poorly understood. There is no consensus on the optimal treatment for this condition.
